# Co-Administration of Anticancer Candidate MK-2206 Enhances the Efficacy of BCG Vaccine Against *Mycobacterium tuberculosis* in Mice and Guinea Pigs

**DOI:** 10.3389/fimmu.2021.645962

**Published:** 2021-05-27

**Authors:** Rania Bouzeyen, Saurabh Chugh, Tannu Priya Gosain, Mohamed-Ridha Barbouche, Meriam Haoues, Kanury V. S. Rao, Makram Essafi, Ramandeep Singh

**Affiliations:** ^1^ Institut Pasteur de Tunis, LTCII, LR11 IPT02, Tunis, Tunisia; ^2^ Translational Health Science and Technology Institute, Faridabad, India

**Keywords:** BCG vaccine, Akt inhibitor, innate immunity, apoptosis, tuberculosis

## Abstract

The failure of *M. bovis* BCG to induce long-term protection has been endowed to its inability to escape the phagolysosome, leading to mild activation of CD8^+^ mediated T cell response. Induction of apoptosis in host cells plays an important role in potentiating dendritic cells-mediated priming of CD8^+^ T cells, a process defined as “cross-priming.” Moreover, IL-10 secretion by infected cells has been reported to hamper BCG-induced immunity against Tuberculosis (TB). Previously, we have reported that apoptosis of BCG-infected macrophages and inhibition of IL-10 secretion is FOXO3 dependent, a transcription factor negatively regulated by the pro-survival activated threonine kinase, Akt. We speculate that FOXO3-mediated induction of apoptosis and abrogation of IL-10 secretion along with *M. bovis* BCG immunization might enhance the protection imparted by BCG. Here, we have assessed whether co-administration of a known anti-cancer Akt inhibitor, MK-2206, enhances the protective efficacy of *M. bovis* BCG in mice model of infection. We observed that *in vitro* MK-2206 treatment resulted in FOXO3 activation, enhanced BCG-induced apoptosis of macrophages and inhibition of IL-10 secretion. Co-administration of *M. bovis* BCG along with MK-2206 also increased apoptosis of antigen-presenting cells in draining lymph nodes of immunized mice. Further, MK-2206 administration improved BCG-induced CD4^+^ and CD8^+^ effector T cells responses and its ability to induce both effector and central memory T cells. Finally, we show that co-administration of MK-2206 enhanced the protection imparted by *M. bovis* BCG against *Mtb* in aerosol infected mice and guinea pigs. Taken together, we provide evidence that MK-2206-mediated activation of FOXO3 potentiates BCG-induced immunity and imparts protection against *Mtb* through enhanced innate immune response.

## Introduction

Despite global efforts and advances in healthcare, Tuberculosis (TB) caused by *Mycobacterium tuberculosis* (*Mtb*) is still the leading cause of mortality from a single infectious agent. Globally, 10 million new cases and 1.4 million deaths due to TB were reported in 2019 (1). Bacilli Calmette-Guérin (BCG) is the most widely used and only available prophylactic TB vaccine (2). Although it is effective against the severe forms of disseminated and pulmonary forms of TB in children, BCG confers only limited and variable protection against the disease in adolescents and adults who account for the majority of TB transmission (3). The protection imparted by BCG is in the range of 0% to 80% and is dependent on the individual’s age and incident rates of TB. Mangtani et al., have reported that in a population at high risk for TB, immunization with BCG was able to show protection for at least 10 years. In comparison, in population at low-risk for TB, BCG immunization was able to impart protection for at least 20 years (4). Hence, there is an urgent need for better control and prevention strategies to stop disease transmission and sustain a long-term TB control (5). Several other strategies such as live attenuated, heat killed, viral vector based, protein subunits and DNA vaccines have been employed to develop alternate TB vaccines (6). However, it still remains elusive to replace BCG because of its safety and only few candidate vaccines have significantly showed better protection than BCG in either animal studies or clinical trials (7). Host-directed procedures that amplify the BCG-elicited protective response might prove useful to control disease transmission and enhance the protection imparted by BCG vaccine.

The available data from experimental and clinical studies suggest that an effective immune response against TB relies mainly on a robust effector and memory T cells responses (8). Consequently, an efficient TB vaccine should be able to induce the formation of antigen-specific polyfunctional type-1 CD4 ^+^ and CD8^+^ T cells through peptides presented by MHC-II and MHC-I, respectively, on the surface of infected macrophages (9, 10). The decrease of BCG effectiveness has been endowed to the defect in antigen processing which subsequently results in diminished Th1 responses. It has been demonstrated that immunization with BCG is unable to induce effective CD8^+^ and CD4^+^ T cells responses and this deficiency might be one of the factors accounting for its poor efficacy (11, 12). In fact, BCG bacilli sequester within phagosomes of macrophages and phagocytes subsequently fuse with lysosomes for degradation and generation of peptides (11). Thereafter, peptides bound to MHC-II complex are exported to the plasma membrane for presentation to CD4^+^ T cells. Further, BCG also secretes proteins that are either cleaved within the phagosomes or processed for presentation along with MHC-I to CD8^+^ T cells (11). Previous studies have reported two defects in antigen processing which affects the ability of BCG-infected phagocytes to prime T cells. It has been shown that BCG residing within immature phagosomes might not expose its repertoire of antigens for lysosomes mediated degradation. Moreover, it was reported that limited cleavage of Ag85B and its assembly into MHC-II complex occurs within antigen presenting cells phagosomes (13). This eventually results in inaccessibility of BCG antigens to both the endocytic MHC-II and cytosolic MHC-I antigen presentation pathways and suboptimal activation of T cells and Th1 immunity (14).

It has been reported that apoptosis of infected host cells facilitates mycobacterial antigen presentation to T lymphocytes through MHC-I and CD1 (15). In accordance, vaccination with apoptotic vesicles, purified from BCG-infected macrophages, was able to confer better protection, than BCG, against challenge with *Mtb* in mice. Such higher protection was associated with the enhancement of dendritic cell-mediated cross-priming of CD8^+^ T cells, termed the “Detour pathway” (16). In agreement, it has been demonstrated that the improved vaccine efficacy of the recombinant BCG *ΔureC hly* ^+^ vaccine in comparison to the parental strain was associated with higher cross-priming as a consequence of enhanced apoptosis (17). Furthermore, vaccination with the *ΔsecA2ΔlysA* and *ΔnuoG* mutant strains of *Mtb* also triggered higher levels of apoptosis in comparison to BCG immunized animals and this resulted in better protection against *Mtb* infection (18, 19). Also, immunization with DNA vaccine co-expressing the Ag85A and the human pro-apoptotic caspase 3 increased protection against *Mtb* infection in comparison to immunization with DNA vaccine encoding Ag85A only (20). In comparison to immunization with BCG, rBCG strain expressing the human pro-apoptotic protein BAX also triggered macrophages apoptosis and elicited predominantly a Th1 protective response (21). Taken together, these studies suggest that enhancing apoptosis in host cells, during BCG vaccination, might be useful to improve its efficacy.

Previous studies have shown that IL-10 negatively regulates the immune response to mycobacterial infection as it affects the adaptive immune response by impeding the functions of macrophages and DC (22, 23). In agreement, it has been reported that BCG inhibits the expression of MHC-II through IL-10 induction, therefore affecting the optimal activation of CD4^+^ T cells (22). This impaired activation of CD4^+^ T cells subsequently reduces long term persistence and proliferative activity of CD8^+^ T cells, their distribution from lymph nodes to distant organs and consequently delayed acquisition of immune protection (24). It has been shown that immunization with BCG in IL-10–deficient mice results in stronger DC activation through increased expression of the surface presenting molecules (25). IL-10 also suppresses the innate immune responses of BCG infected macrophages by inhibiting toll-like receptor-mediated signaling and activating the pro-survival pathway PI3K/Akt (26, 27). In concordance, inhibition of macrophages apoptosis by *M. bovis* also correlates with increased IL-10, Bcl2 and decreased TNF-α production (28). Moreover, induction of IL-10 production in both human and mice upon BCG vaccination limits its protective efficacy (29). Interestingly, it was proposed that BCG-induced Th1 responses progressively switches to Th2, however, in the absence of IL-10, the host immunity leans towards Th1 response post BCG vaccination. In agreement with these findings, IL-10 KO mice vaccinated with BCG presented higher numbers of T cells secreting IFN-γ, IL-17, and TNF-α in comparison to BCG vaccinated wild type mice (30). As expected, the blockade of IL-10 signaling during BCG vaccination resulted in enhanced, sustainable Th1 and Th17 responses and better protection in mice infected with *Mtb* (31). Taken together, these observations suggest that inhibition of IL-10 signaling along with induction of apoptosis during BCG immunization might be a promising strategy to drive stronger Th1/Th17 mediated response and promote long-term immunity against TB.

Previously, we reported that both, apoptosis of BCG-infected macrophages and inhibition of the associated secretion of IL-10, relies on activation of FOXO3, a transcription factor negatively regulated by the pro-survival activated threonine kinase Akt (32, 33). Based on these observations, we hypothesize that activation of FOXO3, using an Akt inhibitor, would enhance BCG-induced host cells apoptosis and inhibit IL-10 production, leading to better protection against TB. To test this hypothesis, we assessed the combination of an anti-cancer candidate Akt inhibitor, MK-2206, and *M. bovis* BCG to impart protection against *Mtb* in aerosol infected mice and guinea pigs. We observed that treatment of macrophages with MK-2206 resulted in enhanced BCG-induced apoptosis and inhibition of IL-10 secretion. The administration of *M. bovis* BCG along with MK-2206 inhibitor increased apoptosis of antigen presenting cells (APCs) in lymph nodes. Further, MK-2206 co-administration improved BCG-induced CD4^+^ and CD8^+^ effector T cells responses and enhanced the ability of BCG to induce both effector and central memory T cells. Interestingly, we also found that co-administration of MK-2206 strengthened the protection conferred by *M. bovis* BCG against *Mtb* in aerosol infected mice and guinea pigs. Taken together, we provide evidence that FOXO3 activation by MK-2206 potentiates BCG-induced immunity and protection against *Mtb* through enhanced apoptosis of host cells and abrogation of IL-10 secretion.

## Materials and Methods

### Cell Culture and Infection

The mouse macrophage cell lines J774A.1 (ATCC TIB-67) and RAW264.7 (ATCC TIB-71) were maintained and cultured in DMEM (Hyclone, USA) supplemented with 5% fetal bovine serum (FBS) (Thermo Fisher, USA) (34). For infection experiments, macrophages were infected with a single cell suspension of *M. bovis* BCG Pasteur strain 1173P2, at a multiplicity of infection (MOI) of 1:10 for 3 h. Subsequently, the extracellular bacteria were removed by washing twice with 1× PBS and cells were overlaid with DMEM medium containing MK-2206 (Selleckchem, USA).

### Apoptosis Assay and Measurement of Caspase Activity

Apoptosis assay was performed according to the manufacturer`s recommendation. Briefly, cells were infected with BCG and subsequently treated with different concentrations of MK-2206. At designated time points, cells were harvested, washed with 1× PBS, stained with APC-Annexin V- and 7AAD (BD Pharmingen, US) and apoptosis was analyzed using FACS Canto flow cytometer (BD Biosciences, US). The cytometric detection of activated caspase-3 and caspase-7 in apoptotic cells was assayed using CellEvent™ Caspase-3/7 Flow Cytometry assay kit using 503/530 nm filter (FITC channel) (Thermo Fischer, USA).

### Western Blotting

For immunoblot analysis, macrophages were lysed in 1× Laemmli buffer and the protein concentration was estimated using Bicinchoninic acid protein assay kit (BCA, Sigma Aldrich, USA). For immunoblot analysis, equal amounts of whole-cell lysate were resolved by electrophoresis on 10% SDS-PAGE gel, transferred to PVDF membrane and subsequently probed with the respective antibodies as per manufacturer recommendations. The primary antibodies used in the study were purchased from either Sigma Aldrich, Merck [FKHRL1-D12 (p-FOXO3Thr32), FKHRL- 1 (FOXO3), β-actin or Cell Signaling Technology, USA (Akt and p-AktSer473).

### Ethics Statement

The animal experiments involving mice and guinea pigs were approved from animal ethics committee from Translational Health Science and Technology Institute (THSTI) and International Centre for Genetic Engineering and Biotechnology (ICGEB). The animal experiments were performed as per the guidelines mentioned by the committee for the purpose of control and supervision of experiments on animals.

### Immunization of Mice and Guinea Pigs

For preparation of vaccine stocks, *M. bovis* BCG (Pasteur stain 1173P2) was cultured in Middlebrook 7H9 medium supplemented with 10% oleic acid albumin dextrose, 0.05% Tween 80 and 0.5% glycerol. The cultures were grown till mid-log phase, harvested by centrifugation, washed, resuspended in 1× PBS and stored as 1,000 μl aliquots at −80°C till further use. The bacterial viability was determined by plating 10.0-fold serial dilutions on Middlebrook 7H11 plates and plates were incubated at 37°C for 3 to 4 weeks. For animal studies, MK-2206 was purchased from Merck and formulated in 30% w/v captisol solution (Cydex pharmaceuticals) as per manufacturer recommendations and previous reports (35, 36). Captisol is a chemically modified β-cyclodextrin that enhances safety, stability, solubility and dermal absorption of the drug (37, 38). For mice experiments, BALB/c mice (female, 6–8 weeks) were subcutaneously immunized and divided into the following 4 groups: (i) sham immunized, (ii) vaccinated with 10^6^ CFU of *M. bovis* BCG, (iii) vaccinated with 10^6^ CFU of *M. bovis* BCG followed by administration of MK-2206 (25 mg/kg) at day 2 and day 3 post-immunization at the same site of vaccination and (iv) administration of MK-2206 at day 2 and day 3 along with the adjuvant. Guinea pigs were immunized *via* intradermal route and divided into 3 groups; (i) sham immunized group, (ii) vaccinated with 10^5^ CFU of *M. bovis* BCG and (iii) BCG immunized and boosted with administration of MK-2206, at day 2 and day 3 post-immunization at the same site of vaccination.

### Preparation of Splenocytes and Lymph Nodes Single Cells

For single cell preparation, spleens and inguinal lymph nodes were treated with complete RPMI medium containing 1 mg/mL of collagenase D (Roche) and 10 U of DNase I (Sigma) at 37°C for 30 min. Subsequently, single cell preparation of collagenase treated organs were prepared and filtered through a 70-μm cell strainer. The single cell suspension was centrifuged, washed with 1× PBS (without Ca-Mg), resuspended in RPMI and enumerated using trypan blue exclusion method.

### Flow Cytometry Analysis

For *ex-vivo* apoptosis assay, live cells from lymph nodes (1 x 10^6^) were incubated for 10 min with anti-CD16/CD32 to block nonspecific antibody binding. Subsequently, cells were stained with Annexin V-PE, anti-CD11b-BV510, anti-CD11c-APC for 30 min at 4°C as per manufacturer’s recommendations (BD Biosciences). The antibody-stained cells were washed twice, suspended in FACS staining buffer and subsequently analyzed using flow cytometry (FACS Canto II, BD Biosciences). For T cell responses, single-cell suspension of spleens was seeded at a density of 1 × 10^6^ cells per well in a 48-well plate and stimulated for 48 h with 10 µg/ml Purified Protein Derivative (PPD; Statens Serum Institut). Subsequently, cells were harvested, washed, incubated for 15 min with anti-CD16/CD32 and subsequently stained for extracellular markers with the following anti-mouse antibodies: anti-CD3-FITC, anti-CD4-Percp, anti-CD8-APCH7, anti-CD44-PE and anti-CD62L-BV421 for 30 min at 4°C as per manufacturer’s recommendations (**Supplementary Table 1**).

For intracellular cytokine staining, spleen cells were *ex vivo* stimulated for 48 h with PPD in the presence of Golgi plug containing Brefeldin A (BD Biosciences) for the last 6 h. Following this, cells were harvested, washed, incubated for 10 min with anti-CD16/CD32 and stained for extracellular markers with anti-CD3-FITC, anti-CD4 -Percp, anti-CD8-APCH7 for 30 min at 4°C (BD Biosciences). The intracellular staining of cells was performed using a cytofix/cytoperm kit with anti-TNF-α-BV421, anti-IFN-γ-Pecy7 and anti–IL-17-BV510 (**Supplementary Table 1**). The stained samples were washed twice, resuspended in FACS staining buffer and data was collected by flow cytometry (FACS Canto II, BD Biosciences). The acquired data was analyzed using Flow Jo software.

### Cytokine Assays

Cytokines levels in the supernatants of PPD stimulated splenocytes were quantified using specific ELISA kits (BD PharMingen, USA). The levels of TNF-α, IFN-γ, IL-2, IL-4, IL-10, IL-12, GM-CSF, and IL-5 in filtered supernatants of lung homogenates were measured using Bio-plex pro mouse cytokine Th_1_/Th_2_ assay kit (Bio-Rad laboratories).

### Protection Studies Against Infection With *Mtb*

Prior to aerosol challenge few animals were euthanized at both 3 weeks and 8 weeks post-immunization to check for residual viable BCG bacilli. As expected, we did not observe any bacterial counts in lungs of immunized animals at both time points. For aerosol challenge, *Mtb*, H37Rv was grown till OD_600 nm_ ~ 1.0 and washed twice with 1× PBS. Subsequently, for mice experiment, single cell suspension containing 5 x10^8^ bacilli was prepared in nebulizer. The infection of immunized mice *via* aerosol route using Inhalation Exposure System (Glas-Col, LLC) resulted in implantation of 300 CFU in lungs. In the case of guinea pigs, single cell suspension containing 1 x10^8^ bacilli was prepared in nebulizer. The infection of immunized guinea pigs *via* aerosol route using Inhalation Exposure System (Glas-Col, LLC) resulted in implantation of 100 CFU in lungs. The disease progression was determined at 30- and 75- days post-infection. For CFU determination, lungs and spleens were homogenized in 2 ml of normal saline and 100 μl of 10-fold serial dilutions were plated on MB7H11 medium at 37°C for 3 to 4 weeks. For histopathology analysis of lungs, 10% formalin fixed lung tissues were stained with hematoxylin, eosin and extent of tissue damage was determined by a pathologist as previously described (39).

### Statistical Analysis

Statistical analyses and generation of graphs were performed with GraphPad Prism version 7 (GraphPad Software Inc., USA). Data are expressed as the mean ± standard error of the mean (SEM). Comparison between groups was performed by paired t-test and differences with a *p* value less than 0.05 were considered as statistically significant.

## Results

### MK-2206 Enhances Apoptosis of BCG-Infected Macrophages *In Vitro*

We have previously reported that FOXO3 activation mediates apoptosis of BCG infected macrophages, an important strategy for host to eliminate intracellular bacteria (33). We have also recently reported that pharmacological inhibition of Akt with MK-2206 induces the activation of FOXO3 transcription factor in BCG-infected macrophages. This results in strong inhibition of IL-10 expression through FOXO3 binding on IL-10 promoter (32). Moreover, such negative regulation of IL-10 secretion favored the establishment of strong M1/Th1 immune responses and inhibited the intracellular replication of mycobacteria. In the present study, we first verified whether the MK-2206–mediated activation of FOXO3 could induce macrophage apoptosis. Murine J774A.1 macrophage cell line was treated with different concentrations of MK-2206 and the percentage of apoptotic cells was evaluated at 24 and 48 h post treatment as described in *Materials and Methods*. As shown in **Figure S1A**, treatment of macrophages with MK-2206 resulted in a dose-and time dependent increase in the percentage of apoptosis compared to vehicle-treated controls. Moreover, we observed that MK-2206–induced apoptosis was caspase-dependent, as shown by the increase in expression levels of effectors, caspase 3/7 activity in drug-treated samples (**Figure S1B**). In comparison to DMSO-treated cells, a 4.0-fold increase in caspase3/7 activity was observed in macrophages upon exposure to 10 µM of MK-2206 for 48 h. We also observed induction of apoptosis by MK-2206 was accompanied with decreased expression levels of both p-Akt and p-FOXO3 in a time dependent manner (**Figure S1C**). Taken together, these observations suggest that decreased phosphorylation of Akt in the presence of MK-2206 promotes FOXO3 dependent intrinsic apoptosis in macrophages. Noteworthy, we have also confirmed our previous data regarding the inhibition, by MK-2206, of the BCG-induced secretion of IL-10 in murine macrophages (**Figure S1D**) (32).

**Figure 1:**
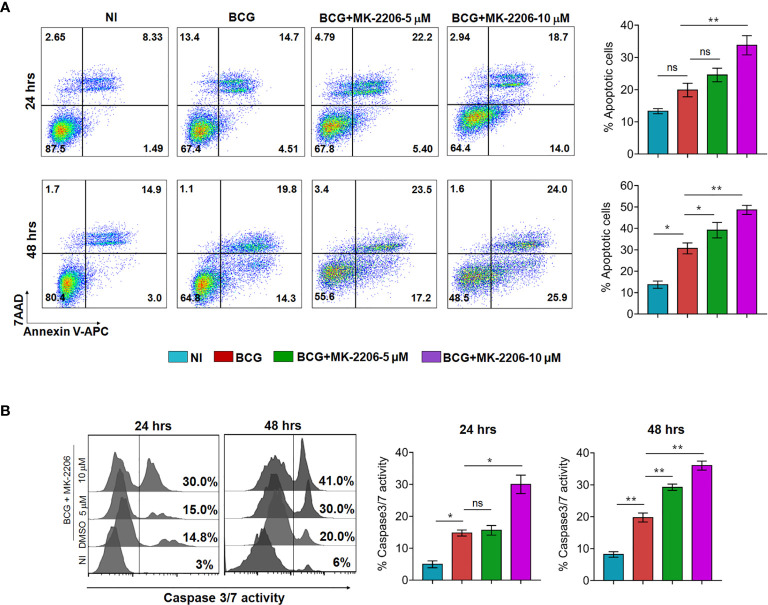
MK-2206 enhances apoptosis in BCG infected macrophages. J774A.1 macrophages were infected with BCG (MOI ~ 10) and subsequently treated with different concentrations of MK-2206 (5 or 10 μM) for 24 and 48 h. Subsequently, macrophages were harvested at the indicated time points and assessed for apoptosis induction and caspase 3/7 activation. **(A)** Representative dot plots show the frequency of apoptotic cells (revealed by the sum of Annexin V^+^/7AAD^+^ cells) at 24 and 48 h post MK-2206 treatment. Histograms depict the percentage of apoptotic cells at 24 and 48 h upon the indicated treatments. The values are presented as mean ± SEM of two experiments performed in triplicates. **(B)** To evaluate caspases activation, cells were stained with 2 μM of cell event caspase-3/7 green detection reagent and the samples were analyzed by flow cytometry. Histograms show representative data from two experiments performed in triplicates. The data shown is mean ± SEM obtained from two experiments performed in triplicates. Statistical differences were obtained between the indicated groups *p < 0.05; **p < 0.01; ns, non-significant; NI, non-infected.

To further verify if MK-2206 treatment enhances apoptosis of mycobacteria-infected macrophages, cells were infected with BCG at MOI of 1:10, treated with MK-2206 and subsequently apoptosis was evaluated. As shown in **Figure 1A**, we noticed that infection did not induce a significant apoptosis compared to uninfected cells at 24 h post-infection. However, the percentage of Annexin V+ increased by approximately 2-fold at 48 h post-infection in comparison to uninfected cells (**Figure 1A**). Furthermore, treatment with the MK-2206 enhanced BCG-induced apoptosis in a dose and time dependent manner upon 24 h (30% and 42% increase of Annexin V+ cells for 5 and 10 μM of MK-2206, respectively) and 48 h (20% and 32% increase of Annexin V+ cells for 5 and 10 μM of MK-2206, respectively) of treatment, in comparison to BCG-infected cells (**Figure 1A**). We also investigated the relative activation of caspases 3/7 in BCG-infected cells post 24 and 48 h of MK-2006 treatment (**Figure 1B**). We found that treatment with MK-2206 increased the activity of caspases 3/7 in a dose and time dependent manner as compared to BCG infection alone. As shown in **Figure 1B**, we observed 15% and 30% increase of caspases 3/7 activity at 24 h for 5 and 10 μM MK-2206 treatment, respectively. Further, 30% and 41% increase of caspases 3/7 activity was observed for 5 μM and 10 μM of MK-2206 treatment, respectively, at 48 h (**Figure 1B**). Taken together, these results implicate that activation of FOXO3 through Akt inhibition post BCG infection results in enhancement of apoptotic cell death.

### MK-2206 Administration Enhances Apoptosis of BCG-Infected Cells *In Vivo* and Triggers APCs Maturation and Their Trafficking to DLNs

**Figure 2:**
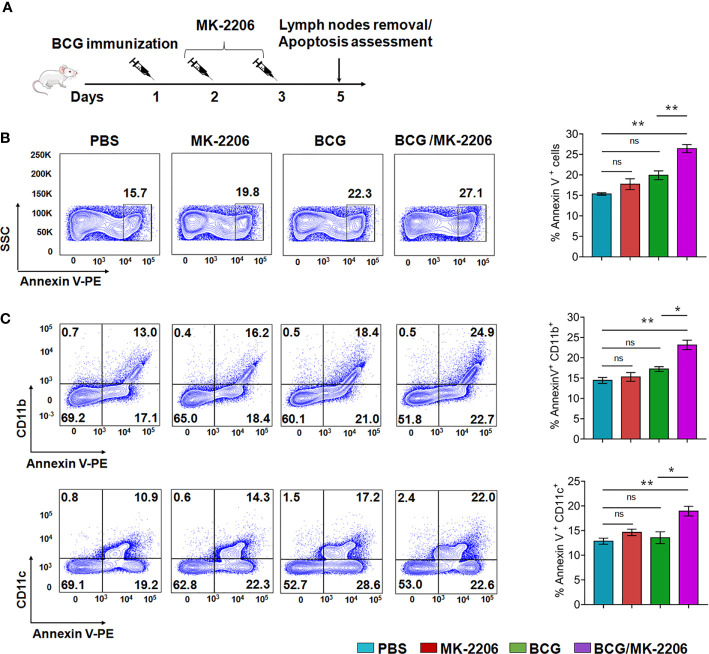
MK-2206 enhances apoptosis in lymph nodes of BCG-vaccinated mice. **(A)** Schematic design of homologous route prime-boost vaccination strategy in mice. Animals were mock treated or primed subcutaneously with 1 × 10^6^ BCG at day 1. Two and three days later, two groups of mock and BCG-vaccinated mice were administered MK-2206 (25 mg/kg) *via* subcutaneous route. Five days after administration, mice (n = 4–5/group) from a single experiment were sacrificed and apoptosis was measured by flow cytometry in draining lymph nodes using Annexin V staining. **(B)** Representative dot plots of apoptotic cells (Annexin V+) in lymph nodes from different groups are shown. **(C)** Frequencies of Annexin V positive cells among CD11b^+^ and CD11c^+^ subsets were measured in DLNs. The data shown in these panels is mean ± SEM of the results obtained from a single experiment (n = 4–5 mice/group). Statistical differences were obtained between the indicated groups *p < 0.05; **p < 0.01; ns, non-significant.

In an attempt to verify the ability of Akt inhibitor, MK-2206, to induce apoptosis of phagocytes *in situ* post BCG immunization, mice were immunized with BCG followed by administration of MK-2206 as described in *Materials and Methods* (**Figure 2A**). It is worth to note that injection of the Akt inhibitor was performed at days 2 and 3 post BCG vaccination to avoid the blockade of mycobacteria phagocytosis, which relies on PI3K/Akt pathway (40). Administration of MK-2206 (25 mg/kg) followed the homologous route of BCG priming in order to target the same DLNs, which has been shown to provide a more effective boost (**Figure 2A**) (41). Following vaccination, BCG resides within mononuclear phagocytes such as dermal macrophages and resident DCs (42, 43). We speculated that the administration of MK-2206 would result in the induction of apoptosis of the infected cells at the local site. Surface exposure of phosphatidylserine molecules in apoptotic cells helps in the efferocytosis of infected apoptotic cells by DCs and macrophages which triggers APCs maturation and their trafficking to DLNs (43). As the first step to elucidate the effect of MK-2206 on apoptosis post BCG vaccination, we performed flow cytometry analysis on lymph nodes cells from the different groups of animals. The subsets of APCs were identified based on the surface expression of CD11c and CD11b using a gating strategy as shown in **Figure S2A**. We found a significant increase in apoptosis at early time points in DLNs of mice vaccinated with BCG/MK-2206 in comparison to sham-immunized and BCG-immunized mice (**Figure 2B**). The induced apoptosis in immunized mice was further characterized in lymph nodes phagocytes using macrophage and dendritic cell-associated markers. As shown in **Figure 2C**, the percentage of apoptotic cells among the CD11b^+^ subsets increased from 18% (BCG group) to 25% (BCG/MK-2206 group), while the frequency of Annexin V^+^ cells among the CD11c^+^ populations increased from 17% (BCG group) to 22% (BCG/MK-2206).

We next checked the infiltration of leukocytes within lymph nodes tissue which were phenotyped and quantified by flow cytometry (**Figure S2A**). CD11c^+^CD11b^+^ were designated as myeloid DCs, CD11c^−^CD11b^+^ were designated as monocytes/macrophages and CD11c^+^CD11b^−^ as recruited macrophages based on previously described functional and morphologic characteristics (44, 45). We observed an enhanced infiltration of CD11c^+^/CD11b^−^, CD11c^−^/CD11b^+^ and CD11c^+^/CD11b^+^ subsets in DLNs of BCG/MK-2206 immunized mice in comparison to BCG-immunized mice (**Figures S2B, C**). These data suggest that administration of MK-2206 in BCG immunized mice triggered the expansion and accumulation of activated and matured APCs in DLNs.

### MK-2206 Augments the Proportion of Multifunctional T Cells in BCG-Vaccinated Mice

**Figure 3:**
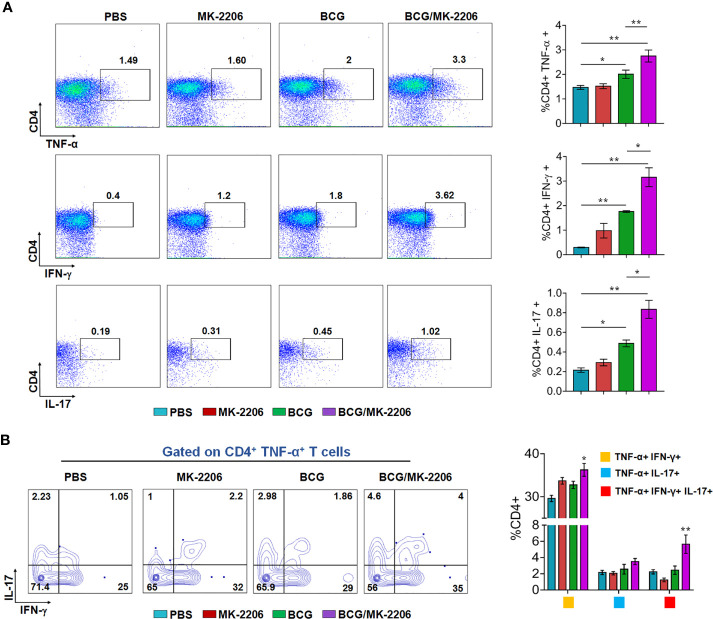
MK-2206 increases Ag-specific multifunctional CD4^+^ T cell responses in vaccinated mice. Each group of mice was vaccinated as described in *Materials and Methods* section. Twenty-one days after immunization, mice from each group (n=5) were euthanized and their splenocytes were incubated *ex-vivo* with PPD for 48 h in the presence of Golgi stop. **(A)** Representative dot plots showing the frequency of PPD-specific CD4^+^ cells producing TNF-α or IFN-γ or IL-17. Data was analyzed by multicolor flow cytometry by gating on CD3^+^CD4^+^ lymphocytes. **(B)** Multifunctional cells were identified by gating on CD4^+^ TNF-α^+^, T cells. Representative FACS plots show the frequencies of CD4^+^ T cells co-expressing TNF-α and/or IFN- γ and/or IL-17. The data shown in this panel is mean ± SEM obtained from a single experiment (n=5 mice/group). Statistical differences were obtained for the indicated groups. *p < 0.05; **p < 0.01.

**Figure 4:**
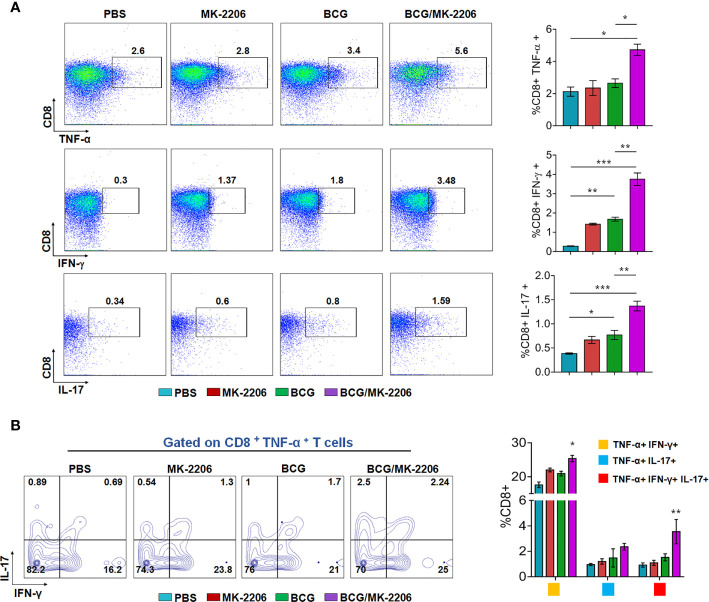
MK-2206 increases antigen-specific multifunctional CD8^+^ T cell responses in vaccinated mice. Each group of mice was vaccinated as described in *Materials and Methods* section. Twenty-one days post -immunization, mice from each group (n=5) were euthanized and their splenocytes were incubated *ex-vivo* with PPD for 48 h in the presence of Golgi stop. **(A)** Representative dot plots showing the frequency of PPD-specific CD8^+^ cells producing TNF-α or IFN-γ or IL-17 were analyzed by multicolor flow cytometry by gating on CD3^+^CD8^+^ lymphocytes. **(B)** Multifunctional cells were identified by gating on CD8^+^TNF-α^+^ T cells: Representative FACS plots show the frequencies of CD8^+^ T cells co-expressing TNF-α and/or IFN- γ and/or IL-17. Results are displayed as mean ± SEM obtained from a single experiment (n = 5 mice/group). Statistical differences were obtained for the indicated groups. *p < 0.05; **p < 0.01; ***p < 0.001.

It is well established that innate responses associated with macrophages apoptosis is associated with an enhanced DC-dependent cross-priming and better antigen-specific CD4^+^ and CD8^+^ T cells in lymphatic tissues (46). Moreover, multifunctional Th1 cells (producing IFN-γ and TNF-α) along with Th17 are known to define a correlate of vaccine-mediated protection (47). Therefore, we next compared the T cell response between BCG/MK-2206 prime boost immunized and BCG immunized mice. As described in *Materials and Methods*, spleens were harvested at 21 days post-immunization and single cell suspension was prepared (**Figure S3A**). Splenocytes were stimulated with PPD and the frequency of CD4^+^ and CD8^+^ T cells producing the signature of cytokines associated with Th1 (IFN-γ, TNF-α) and Th17 (IL-17) was determined by flow cytometry. As shown in **Figure 3A**, we observed that immunization with BCG followed by administration of MK-2206 resulted in significant higher proportion of CD4^+^ T cells producing IFN-γ (100% increase) or TNF-α (65% increase) or IL-17 (127% increase), in comparison to BCG immunized mice. Likewise, we observed that the frequency of CD8^+^ T cells, expressing either IFN-γ or TNF-α or IL-17, was higher in mice primed with BCG and boosted with MK-2206 as compared to mice vaccinated with BCG alone (**Figure 4A**). Further, we characterized the Ag-specific multifunctional T cells based on their ability to produce two or three cytokines. We found that mice vaccinated with BCG followed by MK-2206 administration induced significantly higher multifunctional CD4^+^ T and CD8^+^ T cells in comparison to BCG immunized mice (**Figures 3B**, **4B**). Interestingly, we noticed that multifunctional triple positive T cells (i.e. expressing IFN-γ and TNF-α and IL-17) were prominent in the responding CD4^+^ and CD8^+^ populations in spleens from mice with BCG/MK-2206 prime boost regimen in comparison to BCG immunized mice (**Figures 3B**, **4B**). These results indicate that MK-2206 co-administration during BCG immunization significantly promoted priming of multifunctional T cells and increased Th1 and Th17 protective responses.

### BCG/MK-2206 Prime Boost Regimen Enhances Th1 Responses in Mice

**Figure 5:**
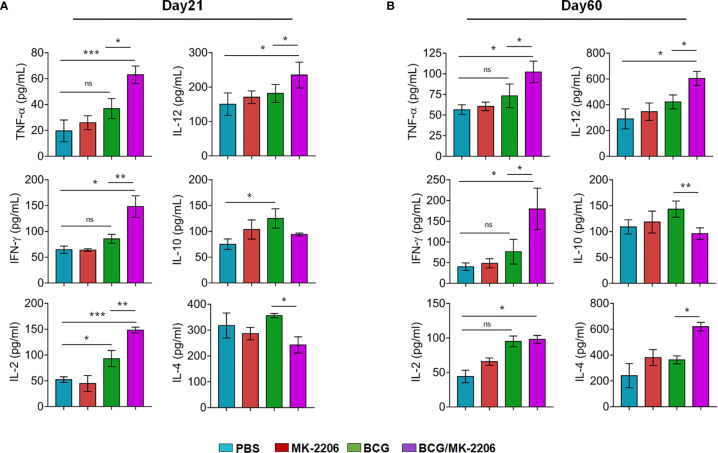
BCG/MK-2206 prime boost regimen enhances Th1 responses in mice. Splenocytes were isolated at 21 days **(A)** and 60 days **(B)** post-immunization and were stimulated *ex-vivo* with PPD for 48 h as described in *Materials and Methods*. The concentrations of representative Th1 cytokines (TNF-α, IFN-γ, IL-2, IL-12) and Th2 cytokines (IL-10, IL-4) were measured in the culture supernatants by ELISA. The data shown is mean ± SEM obtained from a single experiment (n = 5 animals/group). ns, non-significant; Statistical differences were obtained for the indicated groups. *p < 0.05; **p < 0.01; ***p < 0.001. ns, non-significant.

In order to better characterize BCG/MK-2206–mediated increase of specific type 1 responses in vaccinated mice, we performed *ex-vivo* PPD re-stimulations of spleen cells and quantified the levels of the secreted cytokines by ELISA. We observed that cells from mice immunized with the combination of BCG and MK-2206 secreted higher concentrations of pro-inflammatory cytokines such as TNF-α, IFN-γ, IL-12, and IL-2 at day 21 post-immunization in comparison to animals immunized with BCG alone (**Figure 5A**). Concomitantly, by day 60 following immunization, the concentrations of these cytokines were sustained and slightly increased in BCG/MK-2206 immunized mice in comparison to BCG immunized group (**Figure 5B**). The production of Th2 related cytokine such as IL-10 and IL-4 were higher in the PPD stimulated spleen cells from BCG immunized group in comparison to mice immunized with BCG/MK-2206 at 21 days post-immunization (**Figure 5A**). As expected, in comparison to BCG immunized group, a significant decrease in IL-10 production was intensified over 60 days post vaccination in cells from BCG/MK-2206 vaccinated group (**Figure 5B**). However, we observed that IL-4 levels were increased in BCG/MK-2206 immunized group in comparison to the BCG immunized one (**Figure 5B**). Our data suggest that administration of MK-2206 to BCG immunized mice enhanced Th1 response.

### MK-2206 Improves the Memory Responses in BCG-Vaccinated Mice

**Figure 6:**
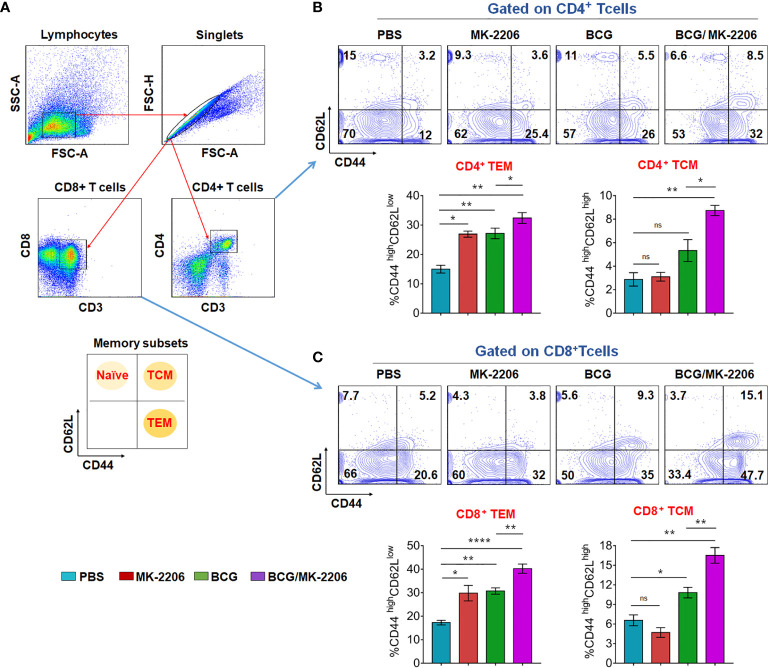
BCG/MK-2206 prime boost regimen induces expansion of effector and central memory T cells. Sixty days post immunization, mice from each group were sacrificed and single cells suspension from spleens were prepared as described in *Materials and Methods*. Splenocytes were stimulated *in vitro* with PPD for analysis of antigen specific memory T cells responses. Live cells were stained with anti-CD3, anti-CD4, anti-CD8, anti-CD44 and anti-CD62L, data was acquired by flow cytometry and subsequently analyzed by FlowJo software. **(A)** Gating strategy used to define CD4^+^ and CD8^+^ memory T cells. Debris were excluded based on FSC and SSC parameters and doublets were excluded in FSC-H versus FSC-A plots. After CD3^+^ gating, the gated populations of CD4^+^ and CD8^+^ T cells were further checked for the expression of memory markers of naïve (CD62L^high^ CD44^low^), central memory (CD62L^high^ CD44^high^) and effector memory (CD62L^low^CD44^high^) T cells. **(B, C)** Representative dot plots showing the percentage of TEM and TCM within CD4^+^ **(B)** and CD8^+^ **(C)** cells. The histograms represent total number of TEM and TCM within CD4^+^ **(B)** and CD8^+^ **(C)** T cells. Data shown in these panels is mean ± SEM obtained from a single experiment (n=5 mice/group). Statistical differences were obtained for the indicated groups. ns, non-significant; *p < 0.05; **p < 0.01; ****p < 0.0001; ns, non-significant.

Long-term vaccine efficacy primarily depends upon the memory response generated during infection (8). Until recently, memory T cells are subdivided into two main subsets, T cells expressing high level of CD62L, termed T central memory (TCM), which reside in lymphoid organs and T cells expressing low level of CD62L named T effector memory (TEM), which survey the site of infection (48, 49). Previous studies have shown that immunization with BCG does not induce sufficient levels of TCM cells in lungs and spleens (50). Moreover, there is a growing evidence regarding the lack of the ability of BCG to protect adults because of its failure to generate long lasting central memory T cells, including CD8^+^ memory T cells (51). Therefore, a strategy that enhances TCM responses would provide improved vaccine efficacy. To elucidate the effect of BCG and MK-2206 combination on memory response, we quantified the proportions of TCM and TEM cells by flow cytometry at 60 days post immunization as per the gating strategy (**Figure 6A**). As shown in **Figure 6B**, we observed that the proportion of CD4^+^ TEM (CD44^high^ CD62L^low^) doubled in mice treated with BCG (from 12% to 26%), compared to sham immunized mice. Interestingly, similar increase was also seen in MK-2206–treated mice in comparison to sham-immunized mice (from 12% to 25.4%). Further, administration of MK-2206 in BCG immunized mice resulted in slight but significant 20% increase in the proportion of CD4^+^ TEM, in comparison to BCG immunized mice. We observed similar findings when we studied the effect of the indicated treatments on the proportion of CD8^+^ TEM (CD44^high^ CD62L^low^). As shown in **Figure 6C**, administration of MK-2206 alone augmented the proportion of CD8^+^ TEM by 35% in comparison to sham-immunized mice. Further, administration of MK-2206 in BCG immunized mice resulted in 27% increase in CD8^+^ TEM levels in comparison to BCG immunized mice (**Figure 6C**). The detailed analysis of TCM cells (CD44^high^CD62L^high^) showed ~42% increase in the proportion of both CD4^+^ and CD8^+^ TCM populations in BCG immunized, in comparison to sham-immunized mice (**Figures 6B, C**). Further, in comparison to BCG-immunized mice, administration of MK-2206 resulted in 36% increase in the number of CD4^+^ TCM (**Figure 6B**). The effect of MK-2206 on BCG-immunized mice was even higher for the proportion of CD8^+^ TCM population resulting in 40% increase (**Figure 6C**). These results indicate that MK-2206 enhances BCG-induced memory responses, especially the pool of TCMs within CD4^+^ and CD8^+^ cells. Based on these observations, we hypothesized that augmentation of TCM and TEM populations, induced by administration of Akt inhibitor in BCG immunized mice might result in better protection against TB.

### Combining MK-2206 to BCG Improves the Vaccine-Induced Protection Against *Mtb* Infection in Mice

**Figure 7:**
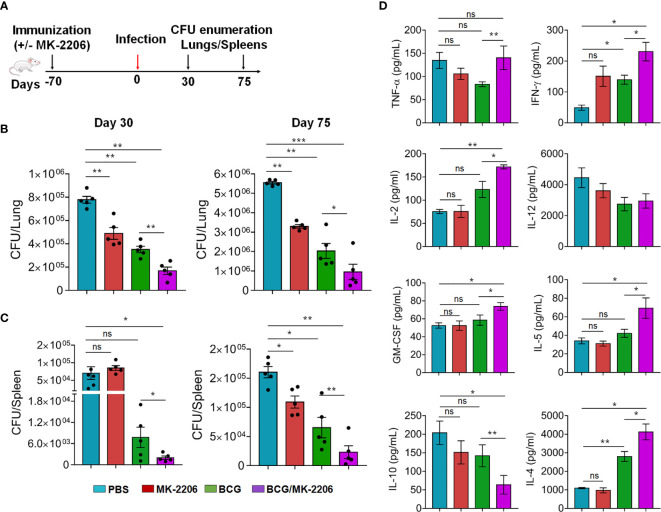
MK-2206 improves BCG elicited protection against TB in mice. **(A)** Schematic representation of protection study in mice. Animals were primed with BCG vaccine and given two boosts with MK-2206 (25 mg/kg) as described in *Materials and Methods*. **(B, C)**. At 70 days post-immunization, animals were infected *via* aerosol route with a high dose of 300 CFU of *Mtb* H37Rv. The lungs **(B)** and splenic **(C)** bacillary loads were determined at day 30 and day 75 post-challenge. For bacterial enumeration, homogenates were diluted 10.0-fold and 100 μl was plated on 7H11 agar at 37°C for 3 to 4 weeks. The data shown in these panels is representative of two experiments and is mean ± SEM CFU obtained from five to six animals per group per time point. **(D)** The levels of cytokines were measured in filtered lung homogenates by Luminex assay. The data shown in this panel is means ± SEM obtained from n = 5–6 mice per group. Statistical differences were obtained for the indicated groups. *p < 0.05; **p < 0.01; ***p < 0.001; ns, non-significant.

In order to assess the impact of MK-2206–mediated enhancement of effective and memory T cells responses on the protective efficacy of BCG, in terms of reduction in bacterial load in mice, we immunized mice as indicated above and determined the bacterial loads over 30 and 75 days post *Mtb* challenge (**Figure 7A**). As expected, at 30 days post infection, the lungs and splenic bacillary loads in BCG-vaccinated mice were significantly reduced by 2.34- and 7.5-fold, respectively in comparison to the sham-immunized mice (**Figures 7B, C**). Similar reduction in bacterial counts was observed at 75 days post *Mtb* challenge. The reduction of bacterial loads was even much higher in lungs (4.8-fold reduction) and spleen (22.5-fold reduction) of BCG/MK-2206 immunized animals, compared to sham-immunized mice (**Figures 7B, C**) at 30 days post challenge. In agreement, the lung and splenic bacillary load was reduced by 9-fold and 6.3-fold, respectively, in BCG/MK-2206 immunized mice compared to sham vaccinated animals at 75 days post challenge. Interestingly, administration of MK-2206 in BCG immunized mice has further augmented the BCG-induced protection by reducing the lung bacillary loads by 2- and 3.5-fold at 30 days and 75 days, respectively, post *Mtb* challenge (**Figures 7B, C**). Moreover, the splenic bacillary load was reduced by 3.0-fold at both time points in BCG/MK-2206 immunized animals as compared to BCG vaccinated mice. It is worth to note, that the sole administration of MK-2206 also reduced *Mtb* burden in the lungs of mice, but with a less pronounced trend in spleens (**Figures 7B, C**).

In order to further characterize the immune responses underlying the better protection observed in BCG/MK-2206 vaccinated mice, we quantified the levels of *in vivo* cytokines in lung homogenates at 75 days post-challenge. We found that vaccination with BCG and co-administration of MK-2206 induced a significant higher magnitude of pro-inflammatory cytokines such as TNF-α, IFN-γ, IL-2, and GM-CSF in comparison to BCG immunized mice (**Figure 7D**). The pro-inflammatory dominant response observed in sham-immunized mice is required for the activation of innate immune cells and increases the recruitment of additional leukocytes into the site of infection for the formation of granuloma and *Mtb* clearance. These observations are consistent with previous reports that showed the importance of Th1 immune response in controlling bacterial infections (47). Concomitantly, IL-10 expression was significantly diminished in mice vaccinated with BCG/MK-2206 in comparison to BCG immunized animals. The observed low expression levels of IL-10 in BCG/MK-2206 vaccinated mice in comparison to BCG immunized mice might explain the improved protection seen in these animals. Paradoxically, we also observed increased expression of IL-4 and IL-5 in the lung homogenates of mice that received BCG/MK-2206 prime boost regimen relative to BCG primed animals (**Figure 7D**). These observations suggest that there is a need to keep a balance between pro- and anti-inflammatory immune responses in order to avoid tissue damage. Since, IL-5 and IL-4 are important for B-cells growth and activation, the observed significant increase in expression of these cytokines in lung homogenates of mice vaccinated with BCG/MK-2206 may reflect a controlled B-cell activation, which might help in controlling *Mtb* infection (52). These observations are in concordance with previous studies suggesting that both cell-mediated and humoral immunity have a role in preventing TB disease (53). 

### MK-2206 Improves BCG Elicited Protection Against TB in Guinea Pigs

**Figure 8:**
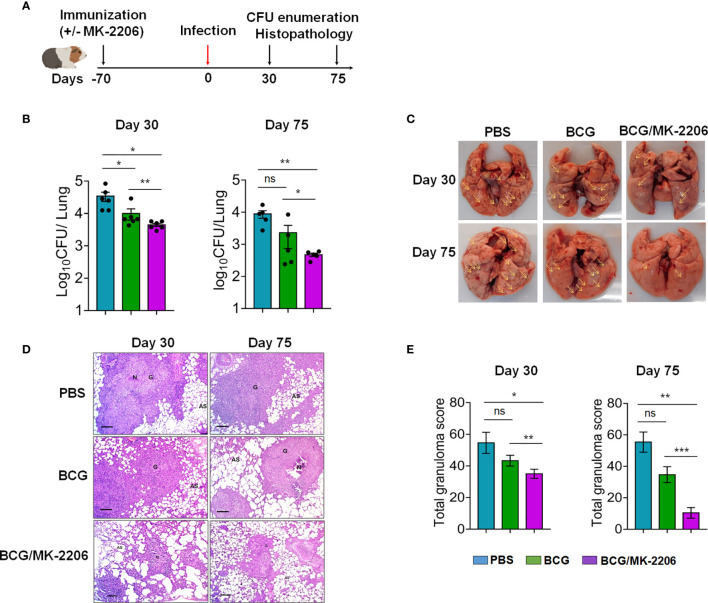
MK-2206 ameliorates BCG elicited protection against TB in guinea pigs. **(A)** Schematic design of protection studies in guinea pigs. The guinea pigs were immunized as described in *Materials and Methods*. After 70 days post-immunization, the animals were infected *via* aerosol route with H37Rv strain **(B)** The lung bacillary load was determined in animals at day 30 and day 75 post *Mtb* challenge. For CFU enumeration, 10.0-fold serial dilution of homogenates are plated on 7H11 agar at 37°C for 3 to 4 weeks. The data shown is mean ± SEM of log_10_ CFU obtained from a single experiment (n = 5–6 animals per group per time point). **(C)** Representative photographs of lung lobes from a representative animal for each group are shown. Yellow arrows highlight the lesions seen in these images as characterized by hemorrhages, swelling or exudative inflammation. **(D)** The section of lungs was stained with Hematoxylin-eosin (HE) and viewed at 40× magnification. A representative section from each group is shown. Scale bar 200 μm. **(E)** The total granuloma score obtained from n = 5–6 animals per group was determined. The data shown in these panels is mean ± SEM of total granuloma score obtained from a single experiment (n = 5–6 animals per group) at day 30 and day 75 post-infection. Statistical differences were obtained for the indicated groups. *p < 0.05; **p < 0.01; ***p < 0.001; ns, non-significant.

We further performed guinea pig experiments to determine whether BCG/MK-2206 can confer a similar level of better protection in comparison to BCG in a more susceptible animal model (54, 55). The guinea pigs were immunized as previously described and animals were challenged *via* aerosol infection that resulted in implantation of 100 bacilli in lungs. Bacterial burdens of each group of animals were assessed over 75 days post-challenge (**Figure 8A**). As shown in **Figure 8B**, vaccination with BCG resulted in 4.5-fold and 2.0-fold reduction of lung bacillary loads at 30 and 75 days post challenge, respectively, in comparison to sham-immunized animals. We observed that co-administration of MK-2206 in BCG immunized guinea pigs resulted in ~ 15.0- fold and 13.0- fold CFU reduction of the viable bacteria in the lungs of guinea pigs at 30 and 75 days, respectively, in comparison to sham-immunized group (**Figure 8B**). We also observed that immunization with BCG followed by administration of MK-2206 significantly reduced lung bacillary load by 5.0-fold at 75 days post-challenge in comparison to BCG immunized group (**Figure 8B**). As shown in **Figure S4A**, we noticed that in comparison to BCG immunized group, animals belonging to BCG-primed MK-2206 group showed reduction in spleen bacillary load by ~ 6.5-fold at 30 days post-challenge. However, no difference has been observed between the splenic bacillary loads in BCG immunized and BCG/MK-2206 immunized groups at 75 days post-challenge. We observed that in both groups, the bacterial loads in spleens of immunized animals were below the limit of detection (**Figure S4A**).

We further substantiated CFU data by evaluating lung pathology and inflammation on histological preparations from different groups. As described in *Materials and Methods*, lung sections were fixed and stained with H&E and the affected area of inflammation relative to the total lung area were analyzed as described previously (56). In sham-immunized and BCG-immunized group, we noticed that *Mtb* infection induced severe pathology that was characterized by the presence of larger size and number of granuloma (**Figures 8C–E**). Importantly, compared to sham- and BCG vaccinated groups, guinea pigs primed with BCG and boosted with MK-2206 had remarkably decreased granuloma like lesions in both lungs and spleens (**Figures 8C** and **S4B**). Interestingly the BCG primed, MK-2206 boosted group exhibited markedly ameliorated histopathology at both 30 days and 75 days post-challenge as manifested by smaller size of granuloma and larger alveolar spaces in comparison to sham- and BCG-immunized animals (**Figure 8D**). In agreement with CFU data, total granuloma score was also reduced by 3.0-fold in BCG-primed MK-2206 group in comparison to BCG only immunized group (**Figure 8E**). These data imply that administration of MK-2206 in BCG immunized animals conferred prevention of severe and extensive lung inflammation compared to that observed in BCG immunized mice after aerosol infection with *Mtb.*

## Discussion

The only available TB vaccine, BCG, provides variable efficacy to pulmonary TB. Therefore, new strategies for the development of a safe and an effective TB vaccine are urgently needed (3). Recent clinical studies on new vaccine candidates for *Mtb* failed to show better protection than the parental vaccine, and a number of these vaccine candidates still use BCG in some form (7). Therefore, development of strategies that enhance the magnitude of existing BCG-mediated protection may overcome the disadvantage of its variable and limited protection (7). One of the key features of an efficient TB vaccine is the priming of antigen-specific T cells to generate memory recall responses, upon encounter with *Mtb* that result in long lasting protection from the infection (9). However, the nature and magnitude of antigen-specific adaptive immune response depends mainly on the baseline immune signature post-vaccination (51). Innate immunity plays a key role in TB vaccine responses and the modulation of innate immune responses upon vaccination results in better protection in animal models (57). Studies have shown that early activation of macrophages upon vaccination, as part of the innate immune mechanisms, contributes to the control of mycobacterial infection. The activation of innate immune response upon BCG vaccination is also essential to shape the subsequent adaptive immune response (29). Moreover, it has been shown that the BCG vaccination site is often prone to an IL-10 driven-Th2 response that inhibits protective Th1, which might be responsible for the failure of BCG vaccine in adults (29).

In the contrary, the blockade of IL-10 following BCG vaccination resulted in an increased Th1, Th17 responses and vaccine mediated-protection against *Mtb* in both susceptible and resistant strains of mice (31). Moreover, apoptosis of mycobacteria-infected phagocytes and efferocytosis promote the recruitment and activation of macrophages and DC which subsequently cross-prime antigen-specific T cells thereby establish protective immune response against aerogenic *Mtb* infection (29, 43). Hence, increasing apoptosis along with the inhibition of IL-10 secretion during vaccination may constitute a promising approach for better priming of the BCG-induced anti-TB immunity. Previously, we have shown that activation of the host FOXO3 transcription factor in mycobacteria-infected macrophages, through the inhibition of Akt by MK-2206, dampened the levels of IL-10 secretion and increased the expression of co-stimulatory molecules in BCG-infected macrophages, leading to a potent M1/Th1 typical immune response (32). Moreover, we have also shown that BCG-induced apoptosis of infected macrophages also relies on FOXO3 activation (33). In the present study, we investigated the impact of FOXO3 activation, using the anticancer candidate Akt inhibitor MK-2206, on BCG-induced immune response and its ability to impart protection against *Mtb* challenge in mice and guinea pigs.

For analysis of Akt inhibition-mediated effects *in vitro*, we investigated the apoptotic functions of MK-2206 using J774A.1 murine macrophage cell line. We observed that inhibition of Akt resulted in activation of endogenous FOXO3 levels and promoted caspase dependent-apoptosis of BCG-infected macrophages. These results are in accordance to previous observations that Akt inhibition promotes the activation of FOXO3 pro-apoptotic functions and increase caspases activity (58). Further studies showed that BCG activates the PI3K/Akt, to gain survival advantages which in turn results in the release of IL-10, inhibition of phagosome maturation and down regulation of antimicrobial responses (59). IL-10 negatively regulates apoptosis *via* STAT3 activation to promote the transcription of anti-apoptotic genes (60). In addition, it has also been demonstrated that transgenic mice expressing human IL-10 under the histocompatibility complex class II promoter, strongly inhibited anti-mycobacterial response of macrophages such as apoptosis and the production of pro-inflammatory cytokines and nitric oxide (61). It has also been reported that induction of cell apoptosis post-BCG immunization resulted in clearance of intracellular bacteria and increased antigen processing leading to improved Th1 responses and better protection (29).

We have previously shown that FOXO3 activation, through inhibition of Akt signaling, at the primary site of infection dampens IL-10 release, increases antigenic presentation of APCs and results in intracellular clearance of BCG (32). Thus, we hypothesized that local administration of apoptosis inducer (FOXO3 activator, MK-2206) at the site of BCG vaccination might result in apoptosis of infiltrating phagocytes and increased protection. A key question in evaluating efficacy of BCG-booster vaccine is when to administer a booster to avoid immunological interference. Here, intradermal vaccination with BCG allowed the phagocytosis of live bacilli before administration of Akt inhibitor at the site of BCG inoculation, as Akt signaling is also involved in the engulfment of bacteria by murine and human phagocytes (62). Moreover, it was previously reported that the homologous route of prime-boost vaccinations targeting DLNs at the local site would provide a more effective boost than the heterologous routes of vaccination targeting distant lymph nodes (41).

We observed co-administration of MK-2206 in BCG immunized mice resulted in a strong influx of DC subsets at early time point in DLNs. Moreover, we noticed that at five days post BCG immunization, numbers of apoptotic cells remained unchanged in DLNs compared to sham-immunized mice. However, the administration of BCG/MK-2206 significantly increased the number of apoptotic cells in DLNs compared to BCG-vaccinated and unvaccinated animals. It is important to note that the administration of MK-2206 alone did not induce cell death and none of the mice died during the experiment or showed any clinical symptoms suggesting the non-toxic effect of the local injection of MK-2206. In addition, we avoided the continuous administration of MK-2206 because Akt kinase has been shown to play an essential role in promoting maturation, migration and survival of APCs (63). Interestingly, Abadie et al., reported that during the first three days post BCG inoculation, phagocytes remained concentrated at the injection site and subsequently these are not observed at early time points in DLNs (42). Our data, suggest that inhibition of Akt promotes DCs activation and their migration to DLNs. This observation is in accordance with previous report showing that the activation of PI3K/Akt pathway upon Leishmania infection subverts DCs immuno-stimulatory abilities and impair the transcription of pro-inflammatory cytokines (64). In addition, it was shown that the inhibition of Akt signaling activates FOXO1, which stimulates the expression of adhesion molecules, ICAM1 and CCR7, required for DCs activation, migration to DLNs and their re-circulation to infected tissues (65). Studies have also shown that IL-10 affects DC trafficking from the site of infection to local DLNs in order to drive polarization of T cells toward Th1 (66). Apoptosis has been described as a defense mechanism against TB infection and has been shown to promote anti-TB protective immunity (67). Indeed, cell death of mycobacteria-infected macrophages deliver antigens to DCs that subsequently cross-prime antigen-specific T cells which subsequently expand and migrate to the lungs to impart protection against *Mtb* infection in mice (68). In the current study, the BCG/MK-2206 boost strategy induced the generation of a high frequency of antigen-specific multifunctional T-cells including IFN-γ, TNF-α and IL-17 triple positive CD4^+^ and CD8^+^ T cells, compared with BCG immunized mice. Previously it has been shown that the magnitude of vaccine-induced polyfunctional T cells have been shown to correlate with vaccine-induced protection against TB (69). Recombinant TB vaccines and subunit vaccines inducing a higher frequency of multifunctional CD4^+^ T cells are shown to confer both short and long term protection against *Mtb* post-challenge (70). However, most protective vaccines elicit a mixture of antigen-specific CD4^+^ and CD8^+^ T cell responses.

The magnitude of CD8^+^ T cell responses is a key immune parameter for optimum control of mycobacterial infection (71). Importantly, the BCG prime/MK-2206 boost approach elicited polyfunctional CD8^+^ T cells producing cytokines, which were amplified in comparison to BCG-immunized group. Presumably, the induction of apoptosis by BCG/MK-2206 vaccine resulted in higher CD8^+^ T cell responses, since enhanced apoptosis is associated with increased cross-presentation of antigens to CD8^+^ T cells and improved immunity against *Mtb* infection *in vivo* (72). Several studies have implicated the involvement of CD8^+^ T cells in protective immunity against TB (24, 73). Indeed, the depletion of CD8^+^ T cells in mice and macaques resulted in failure of BCG to impart protection and increased bacterial burden suggesting that these cells are crucial for control of disease progression (74, 75). In another study, it has been shown that the pro-apoptotic *ΔnuoG Mtb* strain was more effective in priming CD8^+^ T cells in animals and therefore might more effective in clearing intracellular *Mtb* in comparison to the parental BCG vaccine (19).

Our study also revealed that MK-2206 co-administration significantly increased the BCG-induced TCM and TEM pool within CD4^+^ and CD8^+^ T cells. The efficacy of a vaccine relies on the induction of memory responses, which result from the expansion of antigen-specific lymphocytes. Post-infection, TEM appear early at the affected site and have effector functions such as cytokines secretion and provide immediate protection, while TCM home to the DLNs where they proliferate and generate new waves of effector cells upon antigen re-exposure (73). Our results clearly demonstrate that vaccination with BCG/MK-2206 further increased the BCG-elicited protection in lungs of mice and guinea pigs challenged with *Mtb* at 30- and 75 days post-infection. The enhanced efficacy of BCG/MK-2206 in comparison to BCG in mice was associated with an increased expression levels of proinflammatory cytokines such as TNF-α, IFN-γ and IL-2 and a significant decrease in IL-10 production. Among Th1 cytokines, IFN-γ, TNF-α, and IL-2 are classically considered as important components of the antimycobacterial cytokine cascade and are associated with protection against TB (76). These cytokines work synergistically to activate macrophages and maintain the integrity of granuloma (77). The increase in IL-10 levels supports the mycobacterial survival in the host and reduces the protective response to *Mtb* in mice (31). However, the level of IL-4 was increased in BCG/MK-2206 vaccinated mice. These observations are in accordance with previous reports suggesting that IL-4 plays a key role in inducing Th1 cell differentiation by promoting IL-12 and inhibiting IL-10 production by APCs (78). In contrast to the concept that IL-4 acts primarily as an anti-inflammatory molecule, it has been reported that IL-4 has an important role in B cell proliferation, isotype secretion and induction of MHC-II surface expression on these cells, which is important to enhance the activation and recruitment of T cells and eosinophils (79). Moreover, mature Th17 cells are resistant to the effect of IL-4 and are able to secrete IL-17 in Th2 environment (80). The enhanced IL-4 levels in mice immunized with BCG/MK-2206 provides an explanation for the lack of exacerbated inflammation as evident with the reduced immunopathology in the lungs of guinea pigs primed with BCG and followed by MK-2206 administration. These observations suggest that the balance of pro- and anti-inflammatory cytokines plays a critical role in determining the outcome of lung host defenses against *Mtb*. In addition, Th2 cytokines, IL-4 and IL-5 promote B cell class switching to neutralizing antibodies such as IgG1 and they further regulate the magnitude of Th1 cytokines (81). We also found that vaccination with BCG/MK-2206 further increased production of GM-CSF in lung homogenates. GM-CSF overexpression during acute *Mtb* infection contributes to an efficient M1 response, while interfering with GM-CSF pathway might impair the host inflammatory response against *Mtb* (82). This is also in accordance with previous reports showing that GM-CSF based adjuvant formulation is effective to improve BCG immunogenicity (83).

**Figure 9:**
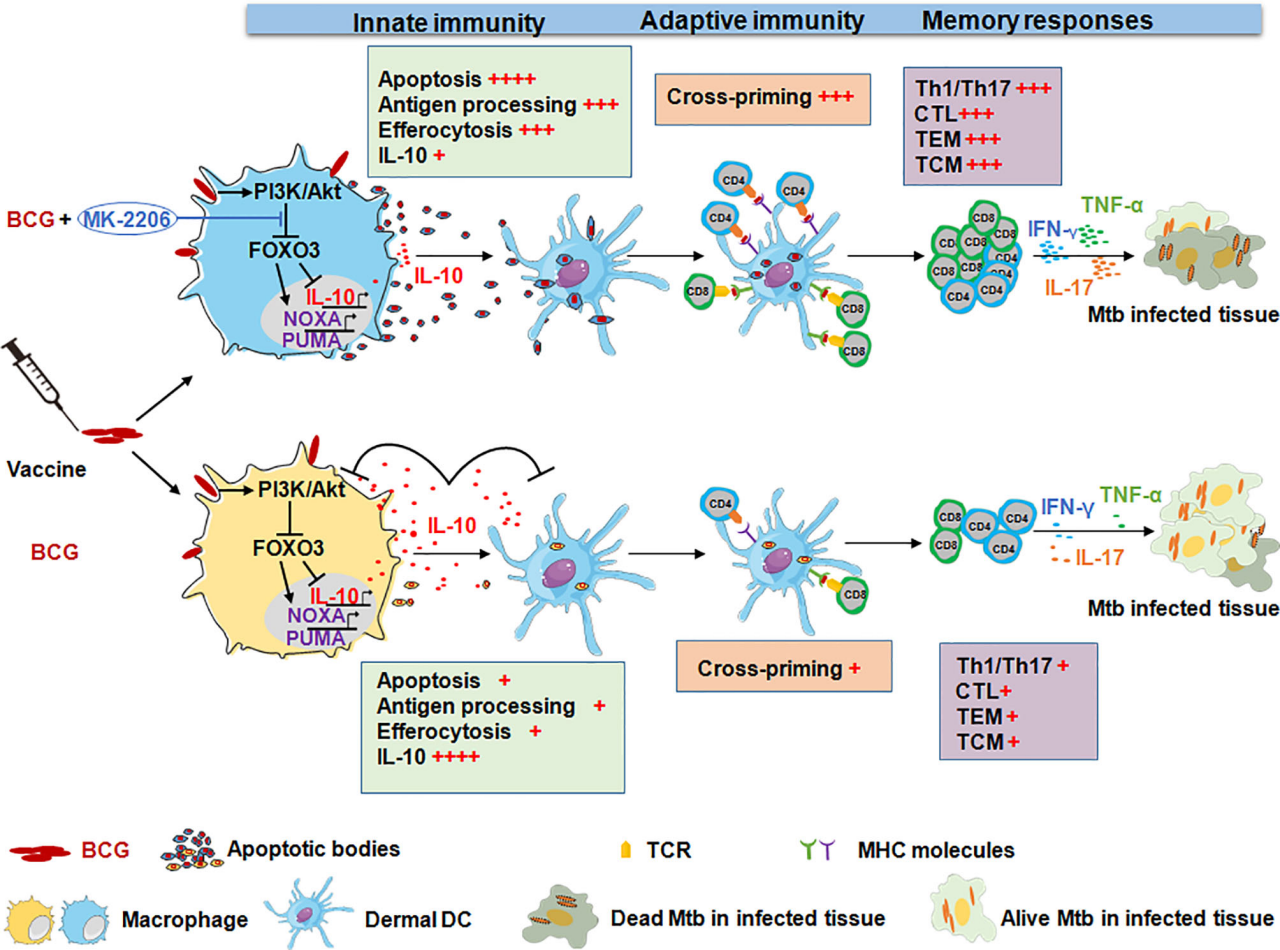
Administration of the Akt kinase inhibitor MK-2206 post BCG vaccination confers higher protection against aerogenic infection. Immunization with BCG induces IL-10 production and also promotes macrophage survival by activating the PI3K/Akt signaling pathway. The activated Akt translocate into the nucleus and phosphorylates FOXO3. The phosphorylation of FOXO3 results in cytoplasmic accumulation and its subsequent degradation. This subsequently results in suppression of Th1 immune response and host antimicrobial pathways, which favors bacterial intracellular replication. In contrast, inhibition of BCG-mediated Akt activation by MK-2206 subsequently results in FOXO3 activation which triggers apoptosis and reduces IL-10 secretion. This eventually results in disruption of growth niche for intracellular replication of BCG, promotes recruitment of APCs and efferocytosis, an important source of antigen presentation. Finally, this leads to optimal induction of antigen specific T cells and the generation of higher memory T cell response that translates into better protection against challenge with *Mtb* in mice and guinea pigs.

In summary, our study reveals that FOXO3 activation, during BCG vaccination, results in induction of apoptosis and a decrease in IL-10 secretion, leading to stronger efferocytosis and higher recruitment of APCs, important sources of Ag for cross-presentation of T cells by DCs. Accordingly, this biological activity induces Ag specific T cells and enhances Th1/Th17 cells, which promotes a robust effective and memory immune response that confer higher protection against *Mtb* infection (**Figure 9**). Our data also support the notion that boosting of the innate immune response further strengthens the BCG-induced protection against *Mtb*. Future experiments would evaluate whether BCG/MK-2206 combination can impart better protection then BCG in Nonhuman primate model. Taken together, enhancing the formulation of BCG vaccine to harness innate immunity offers novel insights into approaches to design vaccines for future TB and non-tuberculous diseases.

## Data Availability Statement

The raw data supporting the conclusions of this article will be made available by the authors, without undue reservation.

## Ethics Statement

The animal study was reviewed and approved by the Translational Health Science and Technology Institute and International Centre for Genetic Engineering and Biotechnology.

## Author Contributions

ME, RB, and RS conceived and designed the work plan. RB, SC, and TG performed experiments. RB, ME, and RS analyzed the data, interpreted them, and wrote the paper as well. M-RB, KR, and MH contributed with providing reagents and discussing the manuscript. All authors contributed to the article and approved the submitted version.

## Funding

The authors acknowledge the funding received from Department of Biotechnology, Govt. of India (BT/IN/Indo-Tunisia/01/2014). This work was supported by the Tunisian Ministry for Higher Education, Scientific Research and Technology (Project Tuniso-Indien session 2013–2016). RB acknowledges Department of Science and Technology India and FICCI for providing C.V. Raman International fellowship. SC acknowledges research fellowship received from Council of Scientific and Industrial Research. TG is also thankful to Department of Biotechnology for her fellowship. RS is a recipient of Ramalingaswami Fellowship and National Bioscience Award from Department of Biotechnology. RS is a senior fellow of Wellcome Trust-DBT India Alliance.

## Conflict of Interest

The authors declare that the research was conducted in the absence of any commercial or financial relationships that could be construed as a potential conflict of interest.
