# Supplementary material for: Co-Administration of Anticancer Candidate MK-2206 Enhances the Efficacy of BCG Vaccine Against Mycobacterium tuberculosis in Mice and Guinea Pigs
Source: Front Immunol. 2021 May 27;12:645962. doi: 10.3389/fimmu.2021.645962 (PMC8190480; doi:10.3389/fimmu.2021.645962)
Supplement: Supplementary file 1 [file DataSheet_1.docx]

**
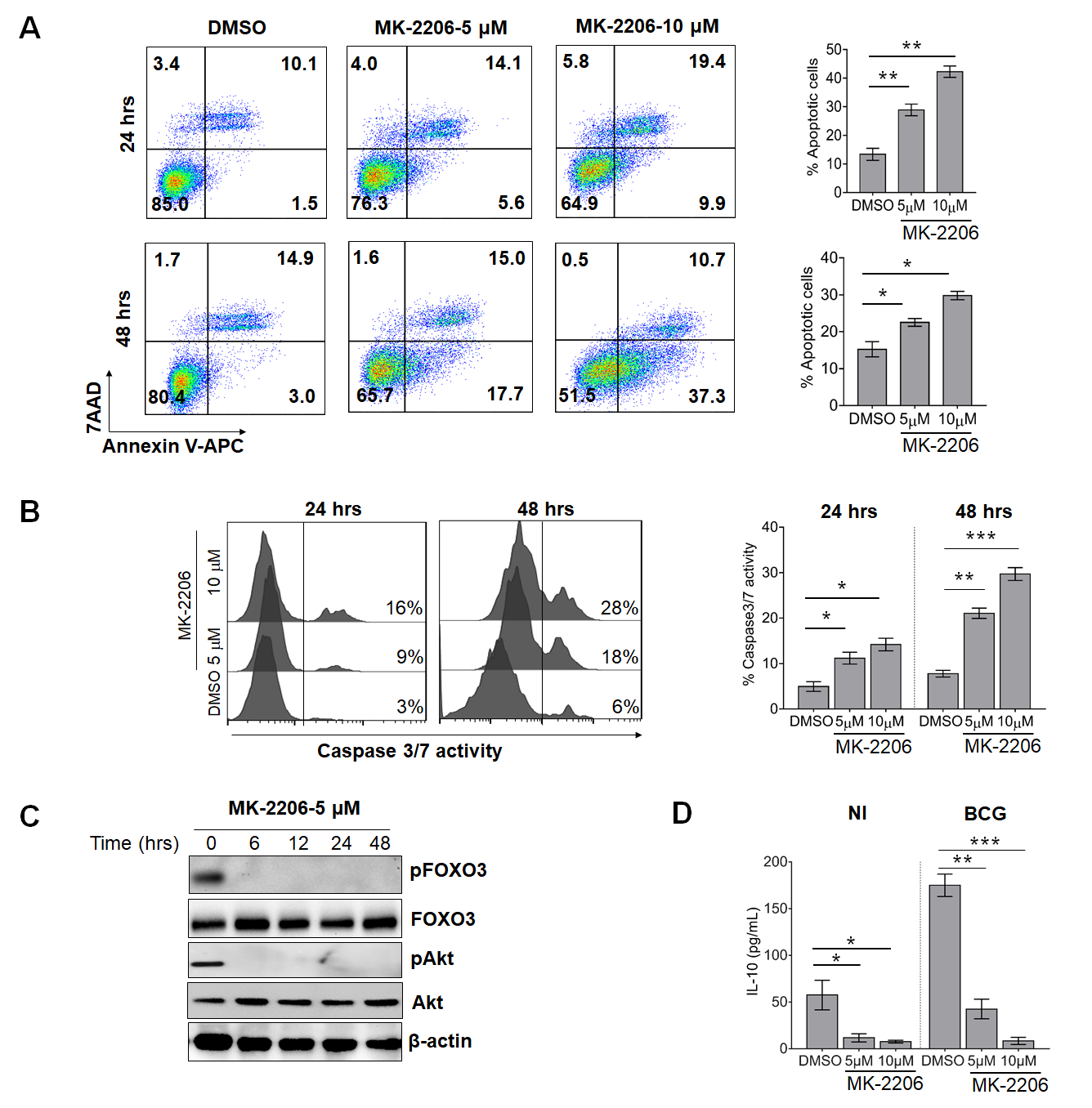
**

**Figure S1: MK-2206 induces macrophage apoptosis through FOXO3 activation:**

J774A.1 macrophage cell line was treated with different concentrations of MK-2206 (5 μM or 10 μM) for either 24 hrs or 48 hrs. The cells were harvested at the indicated time points and assessed for apoptosis induction by Annexin V staining and caspase 3/7 activation. **A.** For apoptosis assessment, cells were harvested at 24 hrs or 48 hrs post-treatment and stained with APC-conjugated Annexin-V and 7AAD (BD Biosciences). The frequency of apoptotic cells was determined by flow cytometry. The histograms depict the percentage of total apoptotic cells at 24 hrs and at 48 hrs post MK-2206 treatment (Right panel). **B.** In order to evaluate caspases activation after MK-2206 treatment, cells were stained with cell event caspase-3/7 green detection reagent and the samples were analyzed by flow cytometry. The histograms depict the percentage of caspase 3/7 activity at 24 hrs and at 48 hrs post MK-2206 treatment **C.** The activities of Akt and FOXO3 were also determined in whole cell lysate of macrophages treated with MK-2206 by Western blotting using the indicated anti-phospho specific antibodies. Total Akt, FOXO3 and β-actin were used as loading controls. **D.** Murine Raw264.7 macrophages were either mock-treated (Non-infected, NI) or infected with BCG (MOI ~ 10) for 3 hrs, washed and treated with Akt inhibitor, MK-2206 (5μM or 10 μM) for 24 hrs. The levels of IL-10 were quantified in culture supernatants by ELISA. The data shown in this panel is mean ± SEM of cytokine levels obtained from two separate experiments performed in triplicates. Statistical differences were obtained between the indicated groups, *p<0.05; ** p<0.01; ***p<0.001.

**
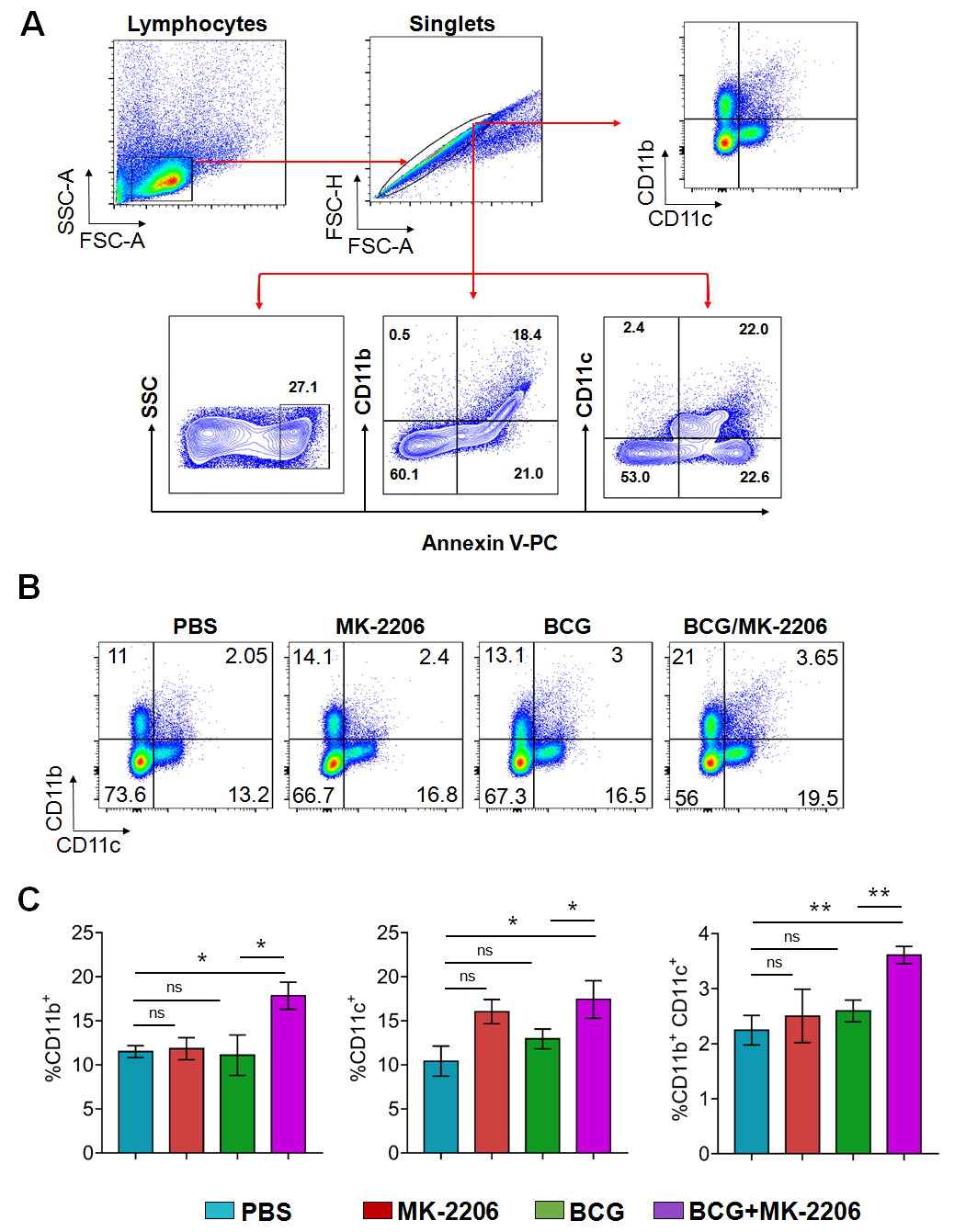
**

**Figure S2: MK-2206 induces the accumulation of DC subsets in DLNs of BCG-vaccinated mice: A.** Flow cytometry gating strategy used for identification of DC and macrophage subsets using panel of surface markers and the induction of apoptosis in lymph nodes from vaccinated mice. Cells were isolated from enzymatically digested mouse lymph nodes and immune cells were stained with anti-CD11b, anti-CD11c mouse antibodies and Annexin V-APC as described in Materials and Methods. **B.** Dots plots showing the frequency of CD11b^+^, CD11c^+^ and CD11b^+^/CD11c^+^ subsets in the DLNs of immunized mice. **(C)** Bar graphs representing the percentage of CD11b^+^, CD11c^+^ and CD11b^+^/CD11c^+^ subsets in the DLNs of mice. The data shown in these panels are represented as mean ± SEM of data obtained from a single experiment (n=4-5 mice). Statistical differences were obtained between the indicated groups. *p<0.05; **p<0.01 ns: non-significant; FSC, forward scatter; SSC, side scatter.

**
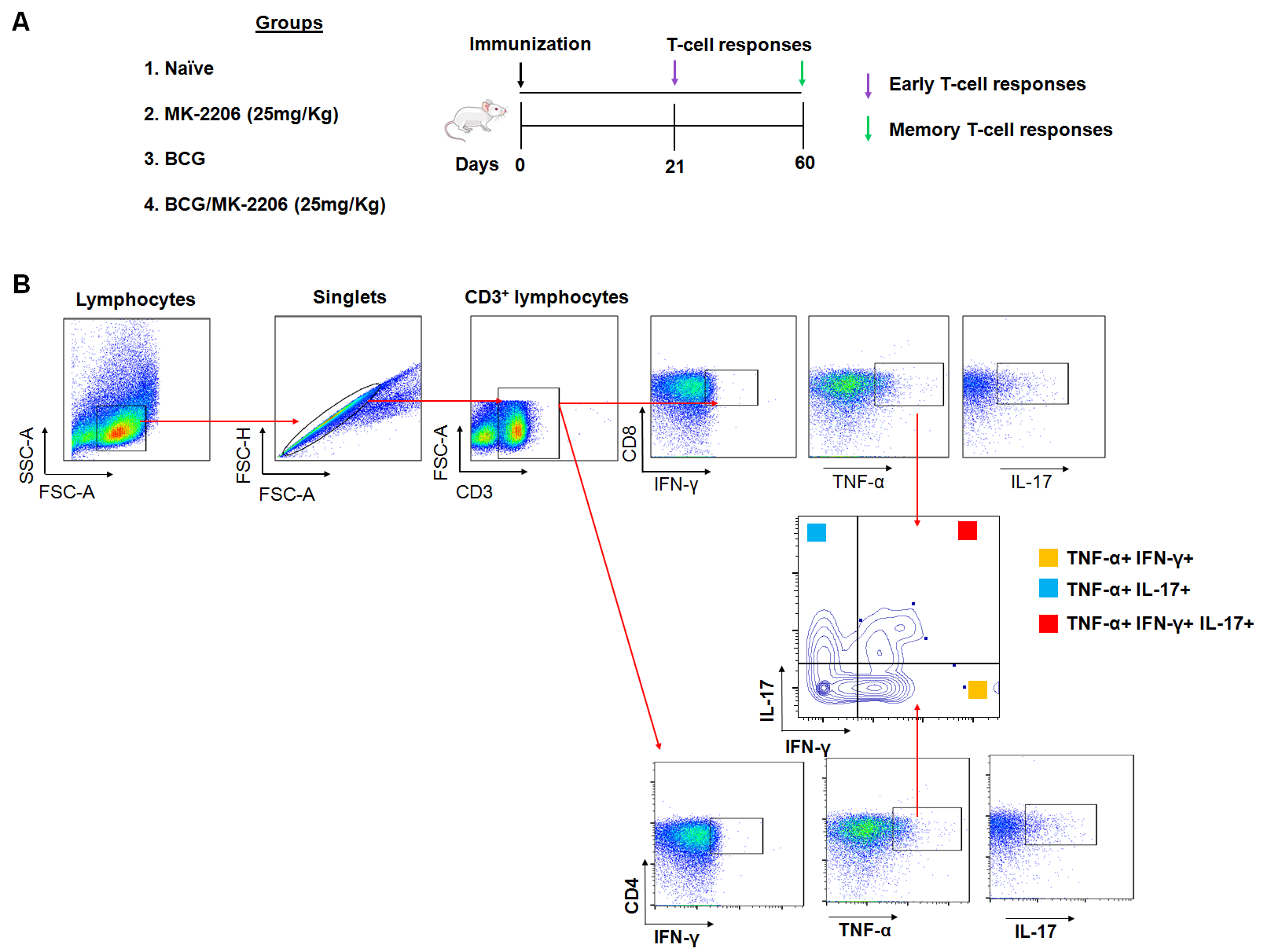
**

**Figure S3: Analysis of multifunctional profile of CD4^+^ and CD8^+^ T cells following BCG/MK-2206 prime boost regimen. A**. Immunization schedule of mice: four groups of mice (n=5/group) were subcutaneously injected with either PBS, MK-2206 (25 mg/kg), BCG or BCG along with MK-2206 (25 mg/kg). Spleens were harvested at 21 days and 60 days post-immunization for analysis of early and memory specific T-cell responses. **B.** Representative flow cytometric analysis showing the gating strategy to identify CD4^+^ and CD8^+^ T cells synthesizing single and multiple cytokines in spleens. After PPD stimulation, live cells were harvested and stained with the following antibodies: anti-CD3, anti-CD4 and anti-CD8. Thereafter, cells were fixed, permeabilized and incubated with anti-TNF-α, anti-IFN-γ and anti-IL-17. The lymphocytes were gated based on cell size (FSC-A versus SSC-A), singlet cells were gated by plotting forward scatter area (FSC-A) versus forward scatter high (FSC-H). CD4^+^ and CD8^+^ T cells were separated in the CD3^+^ population then plotted against each individual cytokine: TNF-α, IFN-γ and IL-17. Analysis was performed with Flow JoV10 software using the combination gate tool in order to obtain single and multi-cytokine-producing cells.

**
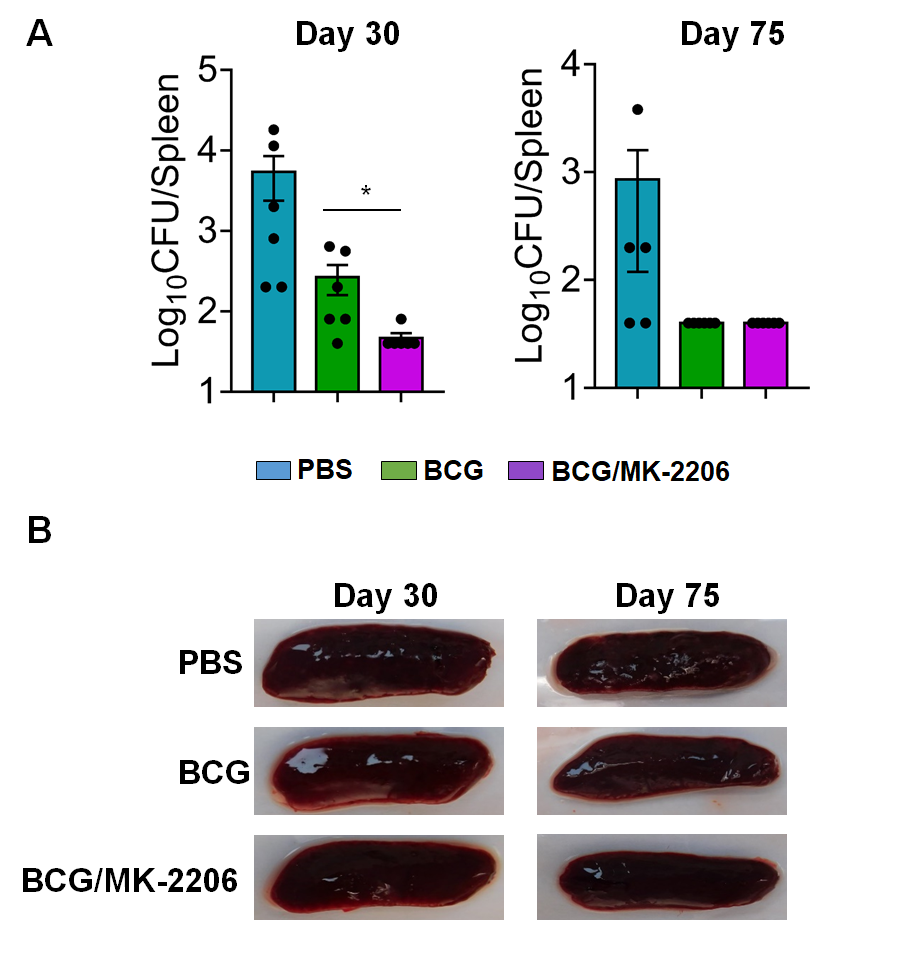
**

**Figure S4. Boosting BCG with MK-2206 during vaccination increases protection to *Mtb* challenge in guinea pigs.** The guinea pigs were immunized as described in section Materials and Methods. After 70 days of immunization, the animals were infected via aerosol route with H37Rv strain. **A.** Splenic bacillary load in animals at day 30 and day 75 post-infection. For CFU enumeration, homogenates were serially diluted and plated on 7H11 agar at 37^o^C for 3-4 weeks. The data shown in this panel is mean + S.E.M of CFU obtained from obtained from a single experiment (n=5-6 animal per group per time point). **B.** The representative photographs of spleen of animals belonging to different groups are shown in this panel. Statistical differences were obtained for the indicated groups, *p<0.05.

**Table S1:** Mouse antibodies and dyes used for flow cytometry listing conjugate, antibody clone supplier and catalog number of each marker.

| Antibody/Dye | Fluorochrome | Clone | Company | Catalog number |
| --- | --- | --- | --- | --- |
| CD11b | BV510 | M1/70 | BD | 562950 |
| CD11c | APC | HL3 | BD | 550261 |
| CD3 | FITC | 17A2 | BD | 561798 |
| CD4 | PerCP | RM4-5 | BD | 553052 |
| CD8 | APC-H7 | 53-6.7 | BD | 560182 |
| CD44 | PE | IM7 | BD | 553134 |
| CD62L | BV421 | MEL-14 | BD | 562910 |
| IFN-γ | PE-Cy7 | XMG1.2 | BD | 557649 |
| TNF-α | BV421 | MP6-XT22 | BD | 563387 |
| IL-17 | BV510 | TC11-18H10 | BD | 564168 |
| Annexin V | PE | - | BD | 560930 |
| 7AAD | - | - | BD | 559925 |
| Annexin V | APC | - | Biolegend | 640941 |
| Cell event Caspase-3/7 Green reagent | - | - | Thermo Fisher | R37111 |
